# Diagnostic efficacy of smear cytology and Robinson’s cytological grading of canine mammary tumors with respect to histopathology, cytomorphometry, metastases and overall survival

**DOI:** 10.1371/journal.pone.0191595

**Published:** 2018-01-23

**Authors:** Izabella Dolka, Michał Czopowicz, Anna Gruk-Jurka, Agata Wojtkowska, Rafał Sapierzyński, Piotr Jurka

**Affiliations:** 1 Department of Pathology and Veterinary Diagnostics, Faculty of Veterinary Medicine, Warsaw University of Life Sciences (SGGW), Warsaw, Poland; 2 Laboratory of Veterinary Epidemiology and Economics, Faculty of Veterinary Medicine, Warsaw University of Life Sciences (SGGW), Warsaw, Poland; 3 Department of Small Animal Diseases with Clinic, Faculty of Veterinary Medicine, Warsaw University of Life Sciences (SGGW),Warsaw, Poland; Colorado State University, UNITED STATES

## Abstract

Cytology is a simple, rapid, and inexpensive method used for pre-operative diagnosis of canine mammary tumors (CMTs) in veterinary practice. Studies related to human breast cancer showed the Robinson’s grading system—established for invasive ductal carcinoma, not otherwise specified (IDC, NOS) and used on cytological material—to not only closely correspond to the histopathological grading but also be helpful in assessing prognosis and selecting most suitable treatments before surgery. The objectives of this study were: to evaluate the accuracy of cytological diagnosis and cytological Robinson’s grading system compared to the histopathological examination of CMTs; to compare of cytological features and cytomorphometric parameters with tumor behavior, as well as cytological and histological grading; and to determine an association of the Robinson’s grading system and cytological background details with metastases, and patients’ survival. We report substantial diagnostic accuracy in detecting simple types and high grade tumors. Cytological diagnosis of tumor behavior showed relatively low sensitivity and specificity compared to human studies, and this might be caused by the heterogeneous morphology of CMTs. The presence of mucosecretory material and extracellular matrix was not significantly associated with tumor behavior. We report a positive correlation between both grading systems and cytological features (included in Robinson’s grading), the presence of necrotic debris, inflammation, and red blood cells. A negative correlation was determined only for the presence of extracellular matrix. The univariate and multivariate analyses confirmed a significantly higher risk of developing metastasis and shorter overall survival for dogs with tumors of grade 2 or 3 on cytology. In addition, these tumors were the most common cause of CMT-related deaths in dogs. Taken together, our findings suggest that the Robinson’s method of cytological grading applied for malignant CMTs evaluated in cytological smears regardless of tumor type can be adapted to veterinary cytology. Additionally, some background features seem to aid malignancy assessment.

## Introduction

Canine mammary tumors (CMTs) are the most common neoplasms of intact female dogs, with malignant tumors accounting for 50% to 70% CMTs [1–3]. They still remain one of the most important health problems, in veterinary medicine likewise breast cancer in human medicine. Even though histopathological examination is considered a key test in CMT diagnosis, cytological examination (cytology, cytopathology, CP) offers several advantages as it is simple, cost-effective, easy to use, relatively non-invasive, and quick in providing results. The use of the fine-needle aspiration cytology (FNAC) and other cytological techniques in veterinary medicine has substantially increased in recent years. Compared to ample reports concerning diagnostic and prognostic accuracy of cytological examination of human breast cancer (HBC), still relatively little data is available regarding CMTs [4–10]. A large number of studies have confirmed CMTs to show many molecular and clinical similarities with those in HBC and therefore various classification systems of CMTs (for example, histological or grading system) were adapted from human research [11–13]. The Nottingham (also known as Elston-Ellis) modification of the Scarff-Bloom-Richardson grading (SBR) system is recommended for histopathology (HP) in HBC. This modification, whose prognostic value has been previously confirmed, is widely accepted for the histological grading of canine mammary carcinomas [14]. The cytological grading method for HBC was first described by Robinson et al. [15], who established a protocol for cytological grading of the most common type of breast cancer, i.e. invasive ductal carcinoma, not otherwise specified (IDC, NOS), also known as invasive ductal carcinoma of no special type (NST).

The Robinson’s grading system corresponds well with the histopathological grades and is considered a useful pre-operative diagnostic tool which provides valuable information concerning the clinical management plan. It helps selecting patients for neoadjuvant chemotherapy as the primary treatment of HBC and avoiding overtreatment of low-grade tumors [15,16]. The National Institutes of Health (NIH), Bethesda, recommends tumor grading of cytological material be incorporated in cytology reports for prognosis [17]. Some authors have even recommended a cytomorphometric analysis to enrich the grading systems [18]. No studies have, however, been published so far, regarding the application of ‘human’ cytological Robinson’s grading system for CMTs. Hence, in this study we aimed to: 1) evaluate the accuracy of cytological examination compared to histopathology, 2) compare cytological Robinson’s grading with histopathological grading, 3) compare cytological features and cytomorphometric parameters with tumor behavior, as well as with cytological and histological grading system, and 4) determine the association of cytological features and the Robinson’s grading system with the development of metastases and patients’ survival.

## Materials and methods

### Ethical statement

According to the Polish animal law (The Act on the protection of animals used for scientific and educational purposes, which was passed in January 2015 and transposed EU Directive 2010/63/EU into current Polish legislation), the Approval of Animal Ethics Commission was not required. Samples were provided by the Department of Small Animal Diseases with Clinic, Faculty of Veterinary Medicine, Warsaw University of Life Sciences (WULS-SGGW), for diagnosis as a part of therapeutic intervention. Ethical guidelines and animal welfare regulations were strictly respected. Dogs’ owners gave their written or verbal informed consent for the use of their animals’ tissue samples. The use of data from retrospective records for research purposes was allowed by the Division of Animal Pathomorphology at the Department of Pathology and Veterinary Diagnostics and Department of Small Animal Diseases with Clinic, Faculty of Veterinary Medicine, Warsaw University of Life Sciences (WULS-SGGW).

### Data collection

A total of 73 female dogs with mammary gland tumors, in which cytology was followed by histopathological examination, were enrolled in the present study. Samples were surgically collected during routine mastectomy and delivered by veterinary clinical practitioners to the Department of Pathology and Veterinary Diagnostics, Faculty of Veterinary Medicine, Warsaw University of Life Sciences between 2007 and 2014. The following clinical data were collected: age at diagnosis, spay status, breed, tumor size (the largest diameter), tumor location, presence of ulceration, regional lymph node status at diagnosis, presence of distant metastases (thoracic X-ray at diagnosis), clinical stages according to the modified TNM (T–tumor size, N–lymph node metastasis, and M–distant metastasis) staging system as follows: stage I (T1N0M0), stage II (T2N0M0), stage III (T3N0M0), and stage IV (TanyN1M0) [19]. Dogs with distant metastases (clinical stage V, TanyNanyM1) were excluded from the study. At least two-year follow-up information was obtained through telephone interview with animal owners and/or the respective veterinarians. Information about survival, cause of death, development of metastases, and local recurrence was collected. Overall survival (OS) was defined as time from surgery to death or euthanasia due to CMT-related cause or any cause, or end of the follow-up.

### Cytological examination

Adequate cytological samples from 73 dogs were mostly obtained during pre-operative cytological examination. CP was performed from one tumor of each dog, the largest one if a dog had multiple CMTs. Fine-needle aspiration cytology was performed using 22 or 23-gauge needles attached to 10 mL syringes. Three to five air-dried smears were stained with a Giemsa solution. Smears for the examination were selected based on their high quality (smear thickness, morphology of cells, proper staining, etc.) [20]. Firstly, cytological smears were scanned at 10x objective per 10 fields using an Olympus BX43 microscope. Cellularity, presence of clusters and background features were evaluated according to the scoring presented in Table 1.

**Table 1:** Scoring system established for cellularity, presence of clusters and background details for evaluation of cytological samples of CMTs in the present study.

Cytologic features	Score			
	0	1	2	3
**Cellularity**	-	Scant 10–20 cells/HPF	Moderate 20–50 cells/HPF	Abundant > 50 cells/HPF
**Cell dissociation (clusters)**	-	Mostly clusters >5/10HPF, single epithelial cells less than 25% neoplastic cells	Clusters 3–4/10 HPF, single cells 25–75% of neoplastic cells	Clusters 1–2/10 HPF, mostly single cells more than 75%
**Mucosecretory material, foamy macrophages**	Absent/ 1–2 cells/HPF	Mild, 3–4 cells/HPF	Moderate, 5–10 cells/HPF	Abundant, >10 cells/HPF
**ECM**	Absent	Mild	Moderate	Abundant
**Necrotic debris**	Absent	Mild	Moderate	Abundant
**Inflammation**	Absent/single 1–2 cells/HPF	Occasional 3–4 cells/HPF	Moderate 5–10 cells/HPF	Abundant >10 cells/HPF
**RBC**	Absent/single 1–2 cells/HPF	Occasional 3–4 cells/HPF	Moderate 5–10 cells/HPF	Abundant >10 cells/HPF

ECM–extracellular matrix, RBC–red blood cells, HPF–high-power field (with 40x objective, a 10x ocular, FN 22 (field of number of ocular), providing a field area of 0.239 mm^2^.

The background components were as follows: mucosecretory material (light blue amorphous material, foamy macrophages), extracellular matrix (extracellular eosinophilic, osteoid-like material), necrotic cellular debris (fragments of fragile cells, cells without recognizable features, basophilic blue-grey amorphous debris), inflammatory cells (neutrophils), and red blood cells (RBC) [6,9,21–23]. We applied the cytological features of malignancy listed in most cytological studies and textbooks for veterinary cytology: hypercellularity, variable cellular size and shape (pleomorphism; anisocytosis, macrocytosis), variable nuclear size and shape (anisokaryosis, macrokaryosis), increased nuclear-to-cytoplasmic ratio; large, prominent or multiple nucleoli, nuclear molding; chromatin clearing, chromatin clumping; presence of abnormal multinucleated cells, and mitotic figures [21,24,25]. Cellular and nuclear details were carefully evaluated in high-power fields (40x objective, 10x ocular, a field area of 0.239mm^2^) and using a 100x objective lens.

**Fig 1:**
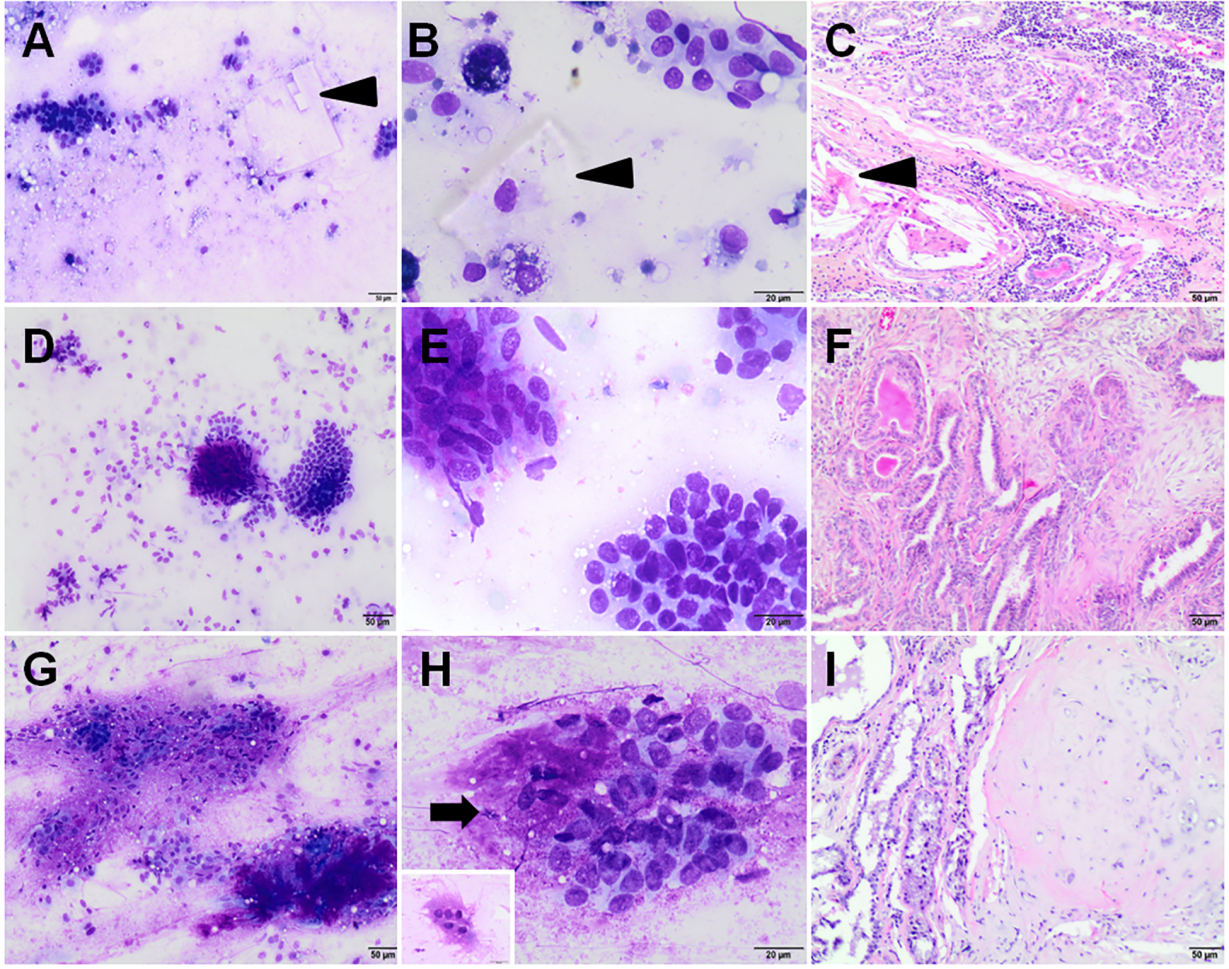
Cytological and histopathological samples of benign CMTs. **Cytological smears were stained with Giemsa, while histopathological samples with hematoxylin and eosin method.** Lobular hyperplasia (A, B–cytology; C–histopathology), arrows indicate cholesterol clefts; Adenoma complex (D, E–cytology; F–histopathology); Benign mixed tumor (G, H–cytology; I–histopathology), an arrow indicates the extracellular matrix, an insert in the bottom-left corner of Fig 1H shows octeoclast. Original magnification: (A, C, D, F, G, I) 100x; (B, E, H) 400x.

**Fig 2:**
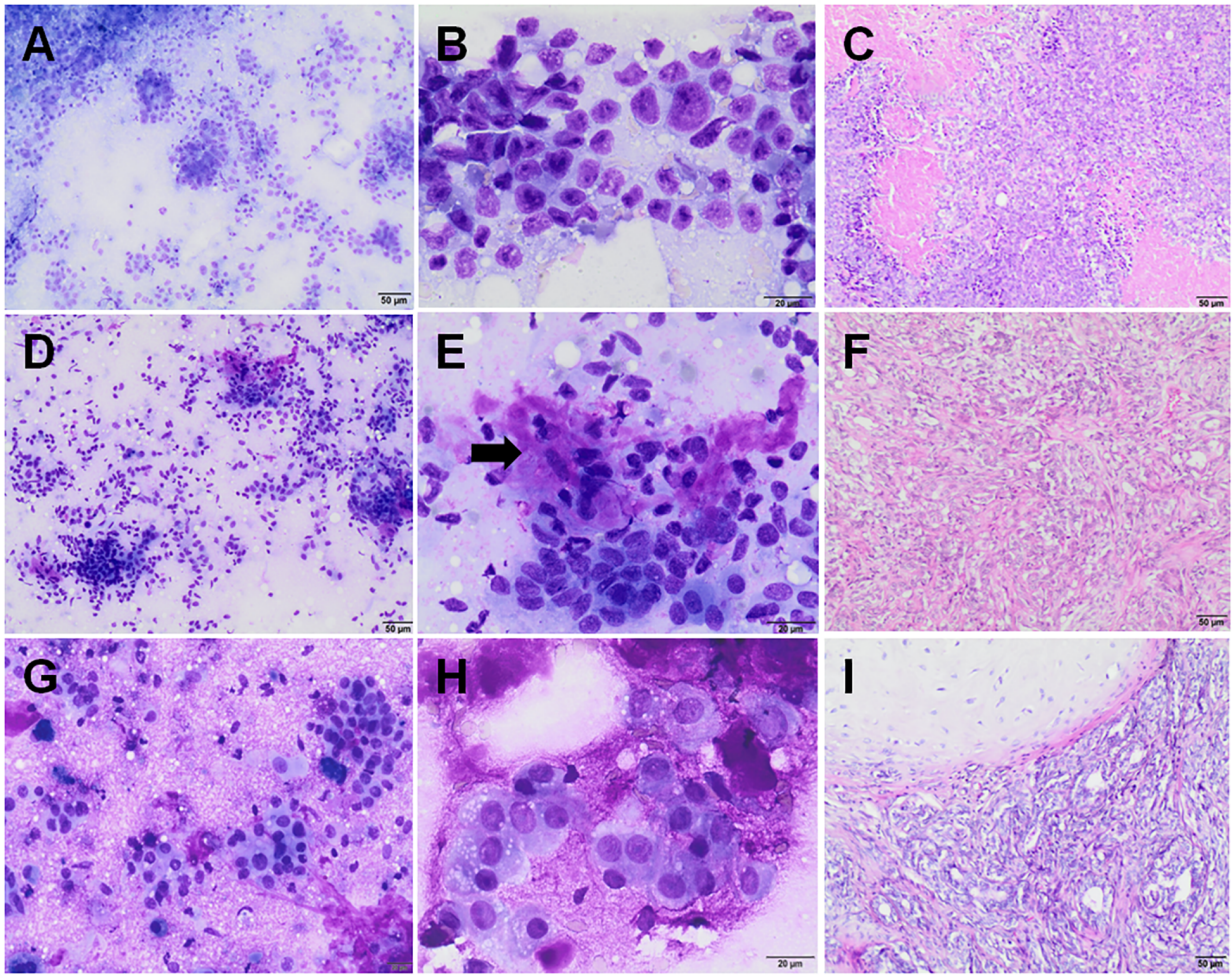
Cytological and histopathological samples of malignant CMTs. **Cytological smears were stained with Giemsa, while histopathological samples with hematoxylin and eosin method.** Simple carcinoma (A, B–cytology; C–histopathology); Complex carcinoma (D, E–cytology; F–histopathology), an arrow indicates the extracellular matrix; Carcinoma arising in benign mixed tumor (G, H–cytology; I–histopathology). Original magnification: (A, C, D, F, G, I) 100x; (B, E, H) 400x.

The samples were classified into two groups: benign tumor (adenoma complex, benign mixed tumor–BMT) and malignant tumor (simple carcinoma, complex carcinoma, carcinoma arising in benign mixed tumor). According to the criteria applied in this study, lobular hyperplasia was added to the group of benign lesions (Figs 1 and 2). Mammary hyperplasia was diagnosed when specimens showed low or moderate cellularity, consisted of normal-appearing mammary epithelial cells (mainly in clusters), eventually with mild atypia; or exhibited a vacuolated cytoplasm (lactational changes). Secretory vacuoles, macrophages, and cholesterol clefts were present (Fig 1A and 1B). The adenoma complex consisted of two cell populations: monomorphic epithelial cells (mostly in clusters) with mild features of malignancy; and cells recognized as myoepithelial cells with spindle to elongated, densely stained nuclei, with moderate or scant pale cytoplasm, or naked bipolar nuclei. Mild amounts of a pink extracellular matrix, and usually mucoid material and macrophages were present as well (Fig 1D and 1E). Cases were considered a benign mixed tumor when the smears were with low/moderate cellularity, contained epithelial cells (with mild pleomorphism) mainly in small clusters; and myoepithelial cells; associated with a large amount of a pink extracellular matrix, and granular eosinophilic background. Occasional osteoclasts might appear as well (Fig 1G and 1H). Samples of simple carcinoma showed moderate/high cellularity; with epithelial cells (single and in clusters) being predominant and showing cytological criteria of malignancy (Fig 2A and 2B). Cytological appearance of complex carcinoma (Fig 2D and 2E) and carcinoma arising in BMT (Fig 2G and 2H) were quite similar, contained many epithelial cells occurring singly and in clusters, with cytological features of malignancy and moderate to large amount of myoepithelial cells, intermixed collagen fibers and a pink extracellular matrix. If the extracellular matrix and eosinophilic background were most abundant, and/or osteoclasts were noted, a sample was diagnosed as carcinoma arising in BMT [23–26]. Cytological results were compared to blinded gold standard histopathology diagnosis.

**Fig 3:**
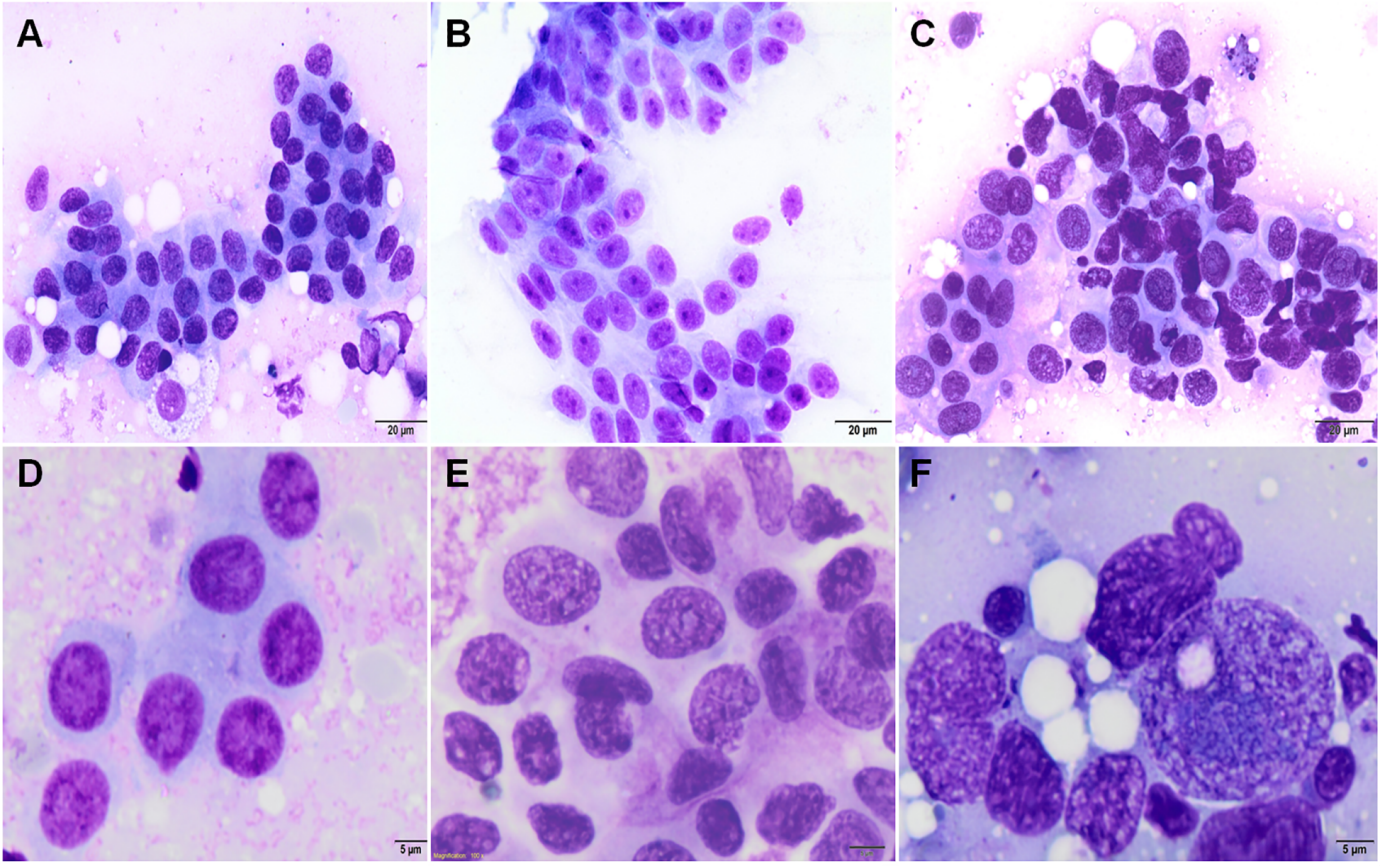
Robinson’s grading system adopted in canine mammary tumors. Robinson’s grade 1. Uniform neoplastic cells in a cluster (A), with a smooth nuclear membrane, vesicular chromatin, and indistinct or nucleoli (D). Robinson’s grade 2. Mildly pleomorphic cells (B), with visible nucleoli, a slightly irregular nuclear margin and granular chromatin (E). Robinson’s grade 3. Pleomorphic cells in a loose cluster (C), with prominent nucleoli, an irregular nuclear membrane and chromatin clearing (F). Giemsa stain. Original magnification: (A, B, C) 400x; (D, E, F) 1000x.

Samples of CMTs cytologically interpreted to be malignant, and stained with Giemsa were evaluated using cytological Robinson’s grading applied in HBC (Table 2) [15]. Six different cytological features were evaluated. The score of 1–3 was given to each of these features, and the tumor was graded by adding the scores together to obtain a total score for each case (Fig 3). Considering the fact that Robinson et al. [15] used wet-fixed Papanicolaou (Pap)-stained smears and only for IDC, NOS, our method should be considered as modified in this respect and adapted for CMTs. The evaluation of nuclear margins in Giemsa-stained smears (although better assessed by staining the smears with Pap test) was supported by computer-assisted nuclear cytomorphometry performed on cytological specimens by outlining nuclei profiles using computation tools.

**Table 2:** Robinson’s grading system described by Robinson et al. [15].

Criterion	Score		
	1	2	3
**Cell dissociation**	Cells mostly in clusters	Mixture of single cells and clusters	Mostly single cells
**Nuclear size**	1–2 times larger than RBC	3–4 times larger than RBC	≥ 5 times larger than RBC
**Cell uniformity**	Monomorphic	Mildly pleomorphic	Pleomorphic
**Nucleoli**	Indistinct/small	Noticeable	Abnormal, prominent
**Nuclear margin**	Smooth	Slightly irregular, folds and grooves	Buds, clefts, tufts
**Chromatin pattern**	Vesicular	Granular	Clumping, clearing, cleaved

Note: Grade 1: 6–11; Grade 2: 12–14; Grade 3: 15–18. RBC–red blood cells

### Cytomorphometric analysis

Cytomorphometry was performed using an Olympus BX43 microscope with CellSens software for image analysis (Olympus, Japan), coupled with a digital camera system. High cellularity fields of neoplastic cells were observed using 40x objective lens, 10x ocular with FN 22 mm. Images were captured and formatted as.jpeg files, and displayed on a computer screen. For each case, 100 cells and 100 nuclei were measured by outlining their profiles using computation tools. Only non-overlapping, non-ruptured nuclei and cells as well as easily detected nuclear and cellular boundaries were analyzed. The nuclei of the epithelial cells were measured in complex and mixed mammary gland tumors. Cellular (mean cellular area–MCA, mean cellular perimeter–MCP, and mean cellular diameter–MCD) and nuclear morphometry were performed (mean nuclear area–MNA, mean nuclear perimeter–MNP, mean nuclear diameter–MND, nuclear roundness–NR), and nuclear to cytoplasmic ratio (N/C) was determined.

### Histopathological examination

Tissue samples were fixed in 10% neutral buffered formalin, routinely processed, embedded in paraffin, sectioned at 4μm, and stained with hematoxylin and eosin (HE). Histopathological diagnosis was performed according to Goldschmidt et al. [11]. The histological tumor grading system of CMTs referred to as Peña system [27] was used, which is based on the assessment of the 3 following morphological features: tubule formation, nuclear pleomorphism and mitotic figures per 10 high-power fields (in the range of 0–0.9; 1.0–1.9; ≥2.0 /HPF). Each of these features is scored with 1 to 3 points. Then, the scores are added to obtain the grade of malignancy, as follows: 3–5 points, grade 1 (low, well-differentiated); 6–7 points, grade 2 (intermediate, moderately differentiated); and 8–9 points, grade 3 (high, poorly differentiated). Fibrosarcoma was assessed according to the grading system for cutaneous and subcutaneous soft tissue sarcoma in the dog [28]. The presence of necrosis, invasive growth pattern, and histological patterns of inflammatory mammary carcinoma (IMC), such as emboli in the dermal lymphatic vessel, were also noted.

### Statistical analysis

Numerical variables were presented as an arithmetic mean and standard deviation (SD) or a median and interquartile range (IQR) unless normally distributed according to the Shapiro-Wilk W test. Numerical variables were compared between two groups with the unpaired-sample Student’s t-test or the Mann-Whitney U test, and with the Kruskal-Wallis test between more than two groups. Categorical variables were presented as a count and percentage in a group and compared between groups with the chi-square test, or the Fisher’s exact test if the expected count in two-by-two contingency table was below 5 (dichotomous variables), and the Mann-Whitney U test, the Kruskal-Wallis test or Spearman’s rank-order correlation coefficient (r_s_) (ordinal variables). Sensitivity (Se), specificity (Sp), and positive and negative likelihood ratio (LR+ and LR-) with 95% confidence intervals (95% CI) and the area under receiver operating characteristic (ROC) curve (AUC) were estimated to assess the accuracy of cytopathology and cytometry versus histopathology as a gold standard. 95% CIs for proportions were calculated using a Wilson score method and for LRs using a log method [29]. The optimal cut-off was identified using a Youden’s J index. The agreement beyond chance between cytopathology and histopathology was determined with a Cohen’s kappa (κ), which was interpreted according to the classification of Landis and Koch [30]. Risk factors of the occurrence of metastases 2 years after the mastectomy were first identified using the univariable logistic regression, or the Mann-Whitney U test (for ordinal categorical variables with more than two categories). Factors for which the p-value was below 0.1 were introduced into the multivariable logistic regression model (as dummy variables if they had more than two categories) according to the backward stepwise procedure to evaluate the role of CP grade as an independent risk factor of the occurrence of metastases 2 years after the mastectomy. Crude odds ratios (OR) and adjusted odds ratios (OR_adjusted_) with 95% CIs were presented to describe the result of the univariable and multivariable analysis, respectively. Prognostic factors of the overall survival (OS) were first identified using the univariable Cox proportional-hazard model (dichotomous and continuous variables) or the generalized Mantel-Cox log rank test (ordinal categorical variables with more than two categories). Factors for which the p-value was below 0.1 were entered into the multivariable Cox proportional-hazard model (as dummy variables if they had more than two categories) according to the backward stepwise procedure to evaluate the role of CP grade as an independent prognostic factor of OS after mastectomy. After surgery, the dogs were followed up for at least 24 months. They were censored if they died from causes unrelated to mammary tumor, or were still alive at the end of the observation period (maximum follow up available 73 months). Crude hazard ratios (HR) and adjusted hazard ratios (HR_adjusted_) with 95% CIs were presented to describe the results of the univariable and multivariable analysis, respectively. The Kaplan-Meier plot and the Mantel-Cox log rank test were used to compare OS between dogs with CP grade 1 tumors and dogs with CP grade 2 or 3 tumors. All statistical tests were two-tailed. A significance level (α) of 0.05 was assumed in all statistical tests except for univariable risk and survival analyses when α was 0.1. The statistical analysis was performed in Statistica 12 software (StatSoft Inc., CA, USA).

## Results

### Patients and tissue samples

Dogs were mostly pure breed dogs (n = 57, 78%), especially small breed dogs (n = 30, 53%): daschunds (n = 9), and Yorkshire terriers (n = 9). The age of all dogs ranged from 5.0 to 15.7 years with the mean (SD) of 10.0 (±2.4) years, and did not differ between pure breed and mongrels (p = 0.799). Ten dogs (13%) were spayed in various times before mastectomy, 58 (79%) had ovariohysterectomy at the same time of mastectomy and, 5 (8%) remained intact. Most of the tumors were located in the 3^rd^–5^th^ mammary gland (n = 64, 91%), slightly more often on the left side (n = 38, 54%). The mean tumor diameter ranged from 0.3 to 20cm with the median of 2.0cm and IQR from 1.5 through 3.5cm. The largest tumors (≥6cm, 5 cases) were noted only on the left side in the 3^rd^–5^th^ mammary gland. Larger tumors (≥ 3cm) were more likely to be malignant, whereas both benign and malignant tumors were present among tumors smaller than 3cm, hence the size <3cm did not allow for any correct classification in terms of tumor behavior. Only 7% of the 27 large tumors (≥3cm) were benign. On presentation, 42 (55%), 16 (22%), 8 (12%), and 7 (11%) tumors were classified as the 1^st^, 2^nd^, 3^rd^ and 4^th^ stage according to the TNM, respectively.

### Accuracy of cytopathology with histopathology in the diagnosis of tumor behavior and tumor type

In histopathology, 18 tumors (24.7%) were classified as benign and 55 (75.3%) as malignant. In cytology, 12 tumors (16.4%) were classified as benign and 61 (83.6%) as malignant (Table 3).

**Table 3:** The numbers and percentages of each CMT based on tumor behavior (benign/malignant) and tumor type determined by cytology (CP) and histopathology (HP, definitive diagnosis) without showing variations in diagnoses between CP and HP (n = 73).

Benign tumors	Cytopathology	Histopathology
	n, (%)	n, (%)
Lobular hyperplasia	2 (16.0)	5 (28.0)
Adenoma complex	5 (42.0)	8 (44.0)
Benign mixed tumor (BMT)	5 (42.0)	5 (28.0)
**Total**	12 (100)	18 (100)
**Malignant tumors**		
Simple carcinoma	28 (46.0)	25 (45.0)
Complex carcinoma	2 (3.0)	12 (22.0)
Carcinoma arising in BMT	31 (51.0)	16 (29.0)
Other (carcinosarcoma, fibrosarcoma)	0 (0)	2 (4.0)
**Total**	61 (100)	55 (100)

In detecting malignant tumors, the cytological examination had Se of 96.4% (95% CI: 87.7%, 99.0%), Sp of 55.6% (95% CI: 33.7%, 75.4%), LR+ of 2.2 (95% CI: 1.3, 3.6), and LR- of 0.07 (95% CI: 0.02, 0.27). The cytological diagnosis of tumor behavior was consistent with histopathology in 63 of all 73 cases (86.3%). Fifty three malignant and 10 benign tumors were diagnosed correctly in CP, while in 2 cases false-negative results (considered as benign with cytology instead of malignant tumor) and in 8 cases false-positive results (considered as malignant with cytology instead of benign tumor) were obtained. For all cases, kappa was 58.5% (95% CI: 35.9%, 81.1%), which was indicative of moderate agreement.

Due to the histological pattern and a low number of some tumor types, for an accuracy analysis of cytological vs. histopathological types, the malignant CMTs were divided into two groups, as follows: 1) simple carcinoma (has only a malignant epithelial component) and 2) complex carcinoma (has a malignant epithelial component and a benign myoepithelial component), carcinoma arising in BMT (has a malignant epithelial component, but the benign component is still detectable–myoepithelial proliferation, foci of cartilage and/or bone), and other malignant tumors (rare mammary tumor types including: carcinosarcoma–composed of a carcinoma and osteosarcoma; and fibrosarcoma–malignant mesenchymal tumor).

A comparison of histological and cytological types of canine mammary tumors is shown in Table 4.

**Table 4:** The comparison of the cytopathological results according to the histopathological diagnosis for the differentiation of benign tumors, simple carcinomas and complex carcinoma/carcinoma arising in BMT/other malignant tumors (n = 73).

CP type	HP type			Total
	Benign tumors	Simple carcinoma	Complex carcinoma/carcinoma arising in BMT/other malignant tumors	
Benign tumors	10	1	1	12
Simple carcinoma	2	22	4	28
Complex carcinoma/carcinoma arising in BMT/other malignant tumors	6	2	25	33
**Total**	18	25	30	73

BMT–benign mixed tumor

In detecting simple carcinoma, the cytological examination had Se of 88.0% (95% CI: 70.0%, 95.8%), Sp of 87.5% (95% CI: 75.3%, 94.1%), LR+ of 7.0 (95% CI: 3.3, 15.1), and LR- of 0.14 (95% CI: 0.05, 0.40). In detecting complex carcinoma/carcinoma arising in BMT/other malignant tumors, the cytological examination had Se of 83.3% (95% CI: 66.4%, 92.7%), Sp of 81.4% (95% CI: 67.4%, 90.3%), LR+ of 4.5 (95% CI: 3.4, 8.5), and LR- of 0.20 (95% CI: 0.09, 0.46). The strength of agreement was substantial (good), with kappa of 65.9% (95% CI: 51.3%, 80.5%).

### Accuracy of cytological vs. histological grading

A comparison of histological and cytological grades is presented in Table 5.

**Table 5:** Benign tumors and malignancy grade classifications (1, 2, 3) of total of 73 CMTs based on cytological vs. histopathological diagnoses.

CP	HP				Total n
	Benign	Grade 1	Grade 2	Grade 3	
**Benign**	10	1	1	0	12
**Grade 1**	8	14	6	1	29
**Grade 2**	0	1	11	1	13
**Grade 3**	0	0	0	19	19
**Total**	18	16	18	21	73

Carcinosarcoma and fibrosarcoma were included (assessed as 3 grade in HP).

The chi-square test revealed a significant association between CP grading and HP grading (p<0.001). Spearman’s rank-order correlation coefficient was 0.863 (p<0.001).

In the analysis of malignant tumors diagnosed in HP as well as in CP, the overall (absolute) concordance rate of Robinson's cytological method (concordance rate = approximate sensitivity) calculated as the percent agreement between cytological and histopathological grading, was 83% and between each CP and HP grade separately was 66.7% for grade 1, 84.6% for grade 2, and 100% for grade 3, respectively. The agreement determined with a kappa value was good (74.5%, CI 95%: 59.9%, 89.2%).

By cytology, 61 CMTs were diagnosed as malignant. There were 8 false-negative cases assigned as grade 1 in CP. The overall concordance rate of Robinson's cytological method was 72.1% (44 out of total 61 were true-positive malignant). As shown in Table 5, most tumors of grade 1 (true-positive cases, n = 14) and grade 3 (true-positive cases, n = 19) were similarly assigned by HP and CP. However, there were also several tumors given a cytological grade of 1 that were diagnosed as benign, or a grade 2 of malignancy on HP (false-positive cases, n = 15). Most tumors given a cytological grade of 2 were similarly graded on HP (true-positive cases, n = 11), but there were also several tumors of grade 2 on HP that received a lower cytological designation (false-negative cases, n = 7). Only two tumors (false-negative cases) that were diagnosed as grade 3 on HP were given a wrong cytological grade, namely a grade 1 or grade 2.

Considering the following reasons: the clinical application of the cytological grading for detecting intermediately (grade 2) as well as poorly differentiated tumors (grade 3), which were associated with similar and worse prognosis than the well differentiated tumors (grade 1) (as further described in the present study), and for having enough cases for meaningful statistical analysis, tumors of grade 2 and 3 were combined into one group. Additionally, this approach was legitimized statistically–significant shortening of the survival time between grade 1 and 2 (p of log-rank test of 0.034) and insignificant between grade 2 and 3 (p of log-rank test of 0.075).

**Fig 4:**
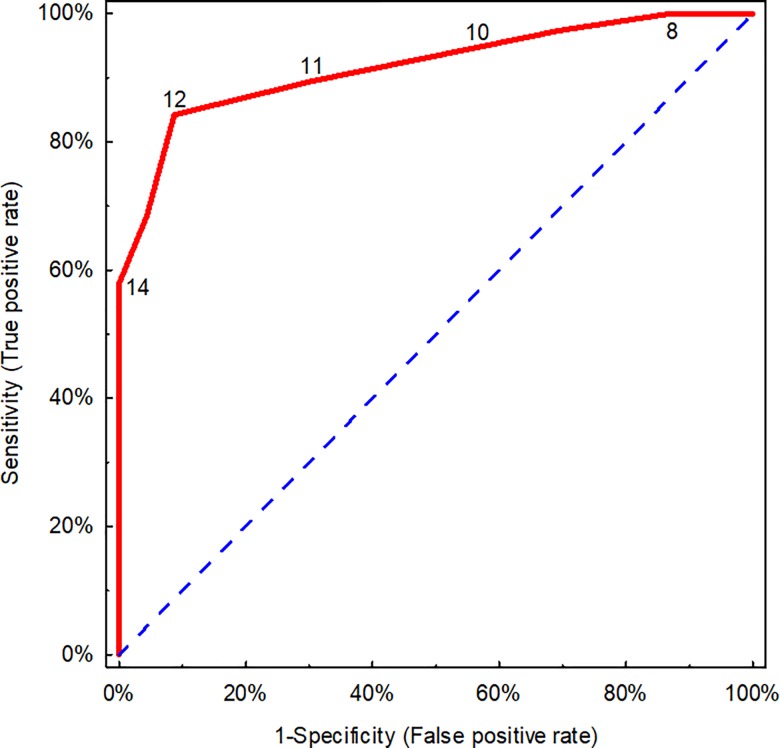
A receiver operating characteristic (ROC) curve of cytopathological Robinson’s grading used for detecting malignant tumors (grade 2 and 3 by HP). Only malignant tumors were included (in cytopathology, n = 61). Number of points on a scale is shown. A broken line is a line of non-discrimination.

We analyzed the accuracy of CP in detecting tumors of grade 2 and 3 by HP. An agreement (a kappa value) between the respective cytological and histopathological grading for tumors of grade 2 and 3 was substantial (good) (75.5%; 95% CI: 60.9%, 90.2%). The Se of cytological examination for grade 2 and 3 was 79.5% (95% CI: 64.5%, 89.2%), whereas Sp was 97.1% (95% CI: 85.1%, 99.5%), LR+ of 27.0 (95% CI: 3.9, 187), and LR- of 0.21 (95% CI: 0.11, 0.39). The area under ROC curve (AUC) was used to show how the Robinson’s grading system performs in distinguishing benign tumors in HP (which had been classified as malignant by CP) or grade 1 in HP from tumors of grade 2 and 3 in HP depending on the number of points in the Robinson’s grading set as a cut-off value. This analysis confirmed that the accuracy of the Robinson’s grading in detecting malignant tumors (grade the 2 or 3 by HP) was 91.7% (CI 95%: 84.8%, 98.6%), p<0.001 (Fig 4) and the optimal cut-off was 12 points according to the Robinson’s grading.

### Comparison of cytological features with tumor behavior, and cytological vs. histological grading system

Most of the cytological features in the cytological samples proved to differ significantly between benign and malignant tumors in CP examination and HP considered as a gold standard (Table 6).

**Table 6:** Cytological features in cytological samples from benign (n = 18) and malignant (n = 55) mammary tumors ultimately diagnosed by histopathology.

Cytological features	Histologically benign tumors n, (%)				Histologically malignant tumors n, (%)				P-value
	Cytological score				Cytological score				
	0	1	2	3	0	1	2	3	
Cell dissociation	-	9 (50.0)	8 (44.4)	1 (5.6)	-	15 (27.2)	20 (36.4)	20 (36.4)	0.013*
Nuclear size	-	10 (55.6)	7 (38.8)	1 (5.6)	-	10 (18.2)	29 (52.7)	16 (29.1)	0.002*
Cell uniformity	-	11 (61.2)	7 (38.8)	0 (0)	-	6 (10.9)	31 (56.4)	18 (32.7)	<0.001*
Nucleoli	-	10 (55.6)	7 (38.8)	1 (5.6)	-	12 (21.8)	27 (49.1)	16 (29.1)	0.004*
Nuclear margin	-	15 (83.3)	3 (16.7)	0 (0)	-	11 (20.0)	24 (43.6)	20 (36.4)	<0.001*
Chromatin pattern	-	17 (94.4)	1 (5.6)	0 (0)	-	16 (29.1)	19 (34.5)	20 (36.4)	<0.001*
Cellularity	-	7 (38.9)	6 (33.3)	5 (27.8)	-	7 (12.7)	15 (27.3)	33 (60.0)	0.007*
Mucosecretory material	1 (5.6)	5 (27.8)	4 (22.2)	8 (44.4)	9 (16.4)	10 (18.2)	17 (30.9)	19 (34.5)	0.478
ECM	3 (16.7)	3 (16.7)	4 (22.2)	8 (44.4)	21 (38.2)	10 (18.2)	10 (18.2)	14 (25.4)	0.058
Necrotic debris	18 (100)	0 (0)	0 (0)	0 (0)	12 (21.8)	11 (20.0)	9 (16.4)	23 (41.8)	<0.001*
Inflammation	10 (55.5)	5 (27.8)	2 (11.1)	1 (5.6)	11 (20.0)	16 (29.1)	17 (30.9)	11 (20.0)	0.003*
RBC	7 (38.9)	6 (33.3)	3 (16.7)	2 (11.1)	3 (5.4)	15 (27.3)	15 (27.3)	22 (40.0)	0.001*

P-value (*) significant at a significance level (α) of 0.05, the Mann-Whitney U test, ECM–extracellular matrix, RBC–red blood cells. Note: refer to Tables 1 and 2 where these features came from.

Malignant tumors received significantly higher scores for all nuclear and cellular features. A background containing necrotic debris, inflammatory cells, and RBC was more often noted in malignant cases. The presence of mucosecretory material and ECM was not significantly associated with tumor behavior.

To determine if there is any reliable correlation between cytological features and grading systems, we decided to include only 44 truly positive malignant CMTs for analysis. All cytological features (cell dissociation, nuclear size, cell uniformity, nucleoli, nuclear margin, and chromatin pattern) assessed in the cytological samples were significantly correlated with both the histopathological and the cytological grading system (p≤0.001 for all). Among background components, the presence of necrotic debris (p<0.001), inflammation (p<0.032), RBC (p<0.027) and ECM (r_s_ = -0.34, p = 0.024) was significantly correlated with CP and HP grades.

### Cytomorphometric parameters in association with tumor behavior, histological and cytological grading

No significant differences were found between cytomorphometric parameters and tumor behavior according to HP diagnosis (S1 Table) as well as both grading systems in the analyzed groups: benign tumors/1 grade tumors vs. 2/3 grade tumors (S2 Table, S3 Table); and tumors of grade 1, 2, 3 in HP (S3 Table), and tumors of grade 1, 2, 3 in CP (S3 Table).

### Cytological grading in association with the occurrence of metastases

Data on the occurrence of metastases were available for 57 dogs 2 years post mastectomy. Fifteen (26.3%) dogs developed metastases to the regional lymph nodes and/or distant organ (lung). Metastases were present in 62.5% (10/16) of tumors of grade 3, 40% (4/10) of grade 2, and 4.5% (1/22) of grade 1 acc. to the Robinson’s system. In the univariable analysis, the occurrence of metastases was significantly associated with parameters provided in Table 7 and S4 Table.

**Table 7:** Univariable analysis of cytological features in association with metastases 2 years after the mastectomy.

Hypothesized risk factor	Category	Metastases 2 years after the mastectomy/ all dogs in the category (%)	Crude odds ratio (95% confidence interval)	P-value
CP grade	-	-	-	<0.001^a^*
CP grade ≥2	No Yes	1 / 31 (3.2) 14 / 26 (53.9)	35 (4.13, 296)	0.001
CP grade = 3	No Yes	5 / 41 (12.2) 10 / 16 (62.5)	12.00 (3.02, 47.61)	<0.001*
Malignant in CP	No Yes	0 / 8 (0) 15 / 49 (30.6)	-^b^	0.068
Simple carcinoma in CP	No Yes	4 / 25 (16.0) 11 / 24 (45.8)	4.44 (1.17, 16.92)	0.023*
Cellularity	-	-	-	0.722^a^
Mucosecretory material	-	-	-	0.200^a^
Extracellular matrix	-	-	-	0.297^a^
Necrotic debris	-	-	-	0.006^a^*
Necrotic debris ≥2	No Yes	3 / 32 (9.4) 12 / 25 (48.0)	8.92 (2.15, 37.08)	0.001*
Necrotic debris = 3	No Yes	5 / 38 (13.2) 10 / 19 (52.6)	7.33 (1.99, 26.97)	0.002*
Inflammation	-	-	-	0.318^a^
RBC	-	-	-	0.109^a^

P-value (*) significant at a significance level (α) of 0.05, a–the Mann-Whitney U test, b–can not be computed when it is 0 or all in one of the category; CP–cytopathology; Necrotic debris ≥2, = 3 –a lesion score of 2 = moderate, 3 = abundant, ECM–extracellular matrix, RBC–red blood cells.

Cell dissociation was not significantly correlated with metastasis (p = 0.088). In the multivariable analysis, CP grade ≥2 remained the only significant risk factor with OR_adjusted_ of 32.50 (95% CI: 3.82, 276.60), p = 0.001.

### Univariate and multivariate survival analysis of dogs with mammary tumors

**Fig 5:**
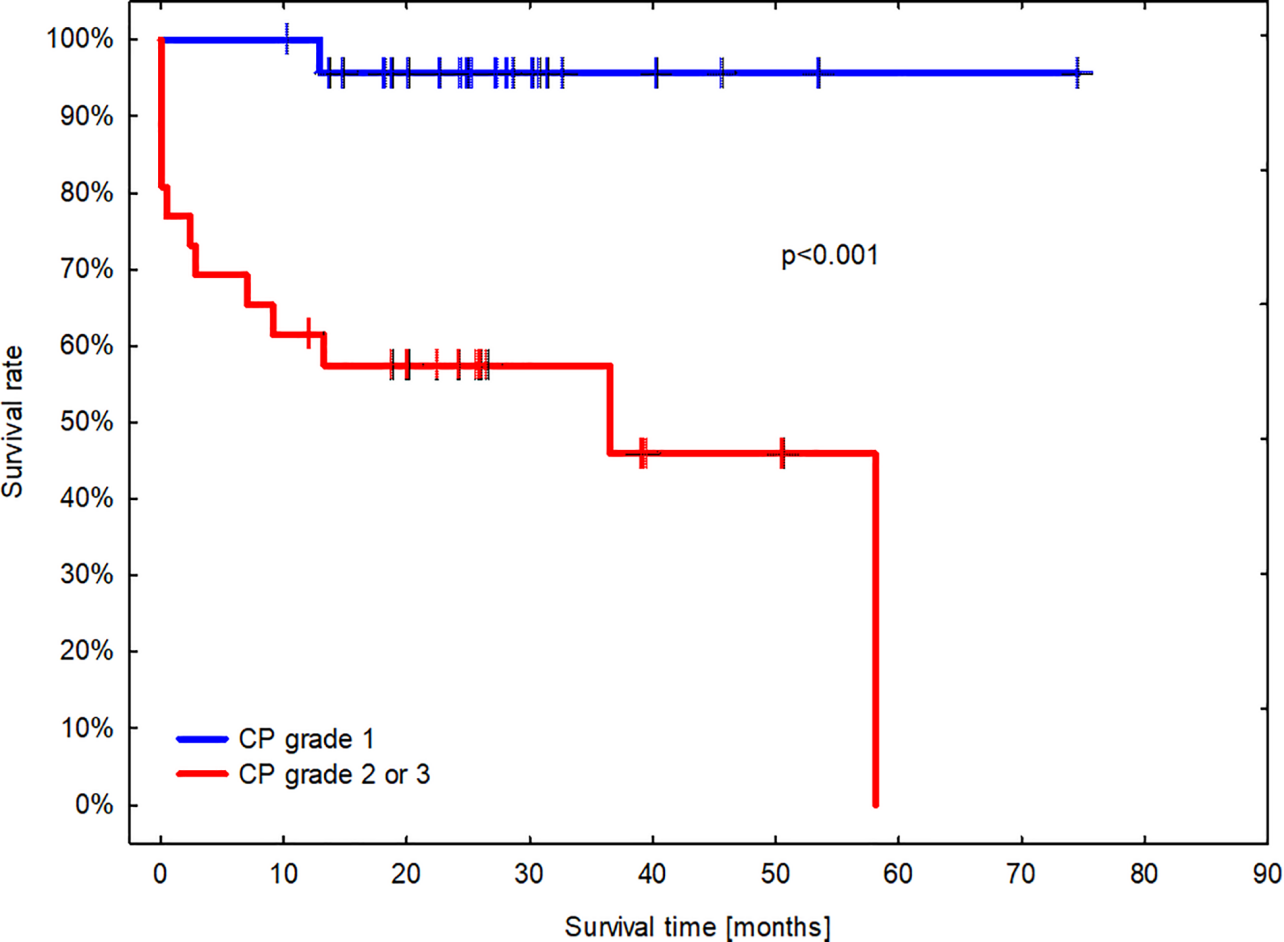
A Kaplan-Meier survival plot of dogs with mammary tumors diagnosed by cytopathology and evaluated with the Mantel-Cox log rank test (α = 0.05). The blue line indicates dogs with tumors of grade 1 (n = 24). The red line indicates dogs with tumors of grade 2 or 3 (n = 26). Short vertical lines signify censored observations. The median overall survival for dogs with mammary tumor graded 2 or 3 by CP was 28 months (IQR 1.5 to 46 months).

Fifty nine dogs were included in the survival analysis. In the follow-up period, 26 dogs died or were humanely euthanized at the owner’s request (44.1%) and 33 (55.9%) stayed alive. Of those which died, 14 (53.8%) died from conditions related to mammary tumor, whereas 12 (46.2%) due to unrelated causes. None of the 9 dogs with benign tumors in CP died. Dogs with malignant mammary tumor in CP had a median survival of 1430 days (48 months). Based on CP, 16.6% (4/24) dogs with tumor of grade 1, 33.3% (3/9) dogs with tumor of grade 2, and 58.8% (10/17) dogs with tumor of grade 3 died during the follow-up period. The median overall survival for dogs with tumor of grade 3 was 305 days (10 months). As revealed in Fig 5, there was a significant difference in survival times between dogs with CMTs based on the cytological Robinson’s grading (p<0.001).

In the univariable analysis, the overall survival regarding CMT-related deaths of dogs was significantly associated with parameters provided in Table 8 and S5 Table.

**Table 8:** Univariable overall survival analysis of dogs with CMTs in relation to cytological examination.

Hypothesized prognostic factor	Category	CMT-related deaths / all dogs in the category (%)	Hazard ratio (95% confidence interval)	P-value
Malignant in CP	No Yes	0 / 9 (0) 14 / 50 (28.0)	-	0.127^a^
Simple carcinoma in CP	No Yes	4 / 27 (14.8) 10 / 23 (43.5)	3.46 (1.08, 11.05)	0.037*
CP grade	-	-	-	0.001^a^*
CP grade ≥2	No Yes	1 / 32 (3.0) 13 / 26 (50.0)	19.58 (2.55, 150)	0.004*
CP grade = 3	No Yes	4 / 42 (9.5) 10 / 17 (58.8)	10.97 (3.00, 40.14)	<0.001*
Cellularity	-	-	-	0.375^a^
Mucosecretory material	-	-	-	0.541^a^
ECM	-	-	-	0.424^a^
Necrotic debris	-	-	-	0.303^a^
Inflammation	-	-	-	0.454^a^
RBC	-	-	-	0.263^a^

P-value (*) significant at a significance level (α) of 0.05, a–generalized Mantel-Cox log rank test. CP–cytopathology; ECM–extracellular matrix, RBC–red blood cells.

In the multivariable analysis, CP grade ≥2 proved to be a significant risk factor with HR_adjusted_ of 9.61 (95% CI: 1.14, 80.77), p = 0.037, along with TNM of 4 which had HR_adjusted_ of 13.29 (95% CI: 3.65, 48.53), p<0.001.

## Discussion

In the pre-operative management of HBC, cytology is an indispensable component of the triple procedure comprising also clinical and imaging examinations. This simple, fast, and non-invasive diagnostic tool is more reliable in diagnosing malignancy, it can also prevent unnecessary surgery, and therefore is widely practiced.

Our results considering CMTs, the breed distribution, the age at diagnosis, tumor size and location were similar to the findings from other studies [1,3,27]. Larger tumors carried a higher risk of malignancy. Similar observations were made in the HBC study where the diagnostic accuracy was higher for larger lesions (over 1 cm) [31].

In previous studies on HBC, the diagnostic accuracy of FNAC in the evaluation of breast lesions compared with histopathology ranged from 90.1% to 95.1%; sensitivity varied from 93.8% to 97.7%, and specificity varied from 80.8% to 100% [31–33]. In CMTs reports, diagnostic agreement of cytological examination for differentiating malignant and benign lesions varied from 63% [34] to 81.4% [8], sensitivity ranged from 25% [21] to 95.23% [5], and specificity from 49% [21] to 96% [9]. Our results are consistent with these from some previous studies [5,8,21,25,35], however provide less favorable results than in human medicine. This could be related to the fact that in our study the false-positive cases were more common compared to the false-negative ones, in contrast to most of the recent HBC studies which show a low false-positive rate of FNAC [36]. It could be due to many factors, such as heterogeneous composition of CMTs which may consist of more than one cell type, a certain degree of pleomorphism observed in the canine benign tumors, the presence of necrosis, irregularly distributed lesions, puncturing the lesion from inappropriate sites, or experience of cytopathologists [25,34,35,37]. On the other hand, we revealed the high sensitivity of CP for malignancy diagnosis, which resulted in the low false-negative rate. CP is rarely expected to be negative in the diagnosis of malignant mammary tumors, thus in such cases therapeutic procedures and further prognosis would be more precise [5]. It is noteworthy that only adequate material with cellular components was included in the current study and this possibly led to the low number of false-negatives in the CP. In contrast, other study on CMTs showed that tumors were more likely diagnosed as benign when they were really malignant [9].

In our study, the overall diagnostic accuracy of tumor types identified cytologically was good. Most agreements were observed among simple carcinomas, while incorrect results among benign tumors, which were misdiagnosed by CP as malignant tumors, i.e. complex carcinoma, carcinoma arising in BMT, carcinosarcomas, and fibrosarcomas. The results are consistent with the previously reported findings, showing that the presence of myoepithelial cells and mesenchymal cells (cells with spindle-shaped appearance) within cytological smears is not limited to one type of CMTs and that their occurrence is not a pathognomonic finding for mixed tumors [38]. Despite known similarities with HBC, mammary mixed tumors are rare in humans and cats but common in dogs. Lale et al. [39] emphasized that metaplastic carcinoma (which refers to a highly heterogeneous group of breast neoplasm with spindle, squamous, osseous, or chondroid differentiation) posed a diagnostic challenge, especially based on cytology. In CMTs, Allen et al. [21] reported spindle-shaped cells in mixed tumors, as well as in myofibroblastomas or spindle cell carcinomas. In our study, these cells were mainly found in complex adenomas, BMT, complex carcinomas, and carcinomas arising in BMT in the cytological samples [25,38]. It could be noted, however, that many of the complex carcinomas, carcinomas arising in BMT were misdiagnosed. As previously suggested [21,25], spindle-shaped cells on CP are attributable to mistakes in differentiation of CMTs, therefore HP should be considered.

To the best of our knowledge, there are no studies addressing the use of the grading system described by Robinson et al. [15] in cytological smears of CMTs and its accuracy with histopathological grading. Various methods were described in cytological grading of HBC, but the Robinson’s cytological grading system has been widely accepted as a simple, more objective and reproducible method, which takes little time and effort, and correlates precisely with the SBR’s histological grading [16,40,41].

In our study, 53 malignant CMTs showed distribution of cytological grades 1, 2, and 3 as 40%, 24% and 36%, respectively, with a slight preponderance of grade 1. In studies carried out on 50 HBCs, 28% cases were cytologically graded as grade 1, 58% as grade 2, and 14% as grade 3 [16], whereas in another study 28%, 48% and 24% cases were graded 1, 2 and 3, respectively [42]. In contrast to our observation, they showed that the majority of cases were in cytological grade 2. Whereas in the study of 608 HBCs Robinson et al. [43] demonstrated that most of the analyzed tumors were in grade 1 and 2 by a similar amount of: 38.3%, 38.5% and 23.2% in cytological grades 1, 2 and 3, respectively.

Investigations on HBC showed a significant association between grades assigned by CP and HP [16,44]. Similar results were demonstrated in our study, which is the first report on CMTs. The agreement (kappa value) between HP and CP grading was substantial (good), which is consistent with previous HBC studies [40,45]. Our results, comparable to these from other studies, showed 83% concordance rate between the cytological Robinson’s method and histological grading for malignant tumors assessed in both HP and CP [46,47]. On the other hand, the concordance rate between grading systems among malignant tumors which were determined by CP was lower by 72.1% in our study. Although this rate was consistent with some previously published reports [41,42,48], it should be noted, that in our study, 8 cases diagnosed as malignant by CP, but benign by HP were included, whereas in the cited reports, only malignant tumors were analyzed. In our study, the highest concordance rate between the Robinson’s grading system and histological grading was found in grade 3, and this finding was comparable with other published data [47]. In addition, the highest degree of concordance between CP and HP grading systems was noted in the high-grade (in grade 3 and then grade 2) compared with the low-grade tumors [42,47]. However, in contrast to our study, some authors have found the highest concordance rate in grade 1 [46,49] or in grade 2 tumors [16,41]. Interestingly, the ROC analysis of the Robinson’s grading showed a high level of accuracy with the gold standard in detecting malignant CMTs of grade 2 and 3. On the other hand, in the present study, all malignant CMTs determined by CP regardless of the histological type were graded according to the Robinson’s method, whereas majority of HBC studies used it for one type of breast cancer [16,44,50]. Therefore, this modification of the Robinson’s cytological grading system with regard to the CMTs should also be taken into account when investigating the causal factors. In the present study, we found the highest number of false-positive cases in the cytological grade 1, and this results in the lowest specificity for grade 1. The reason for that could be the presence of nuclear pleomorphism in the benign tumors that often causes the overdiagnosis of malignancy, or nuclear pleomorphism due to simultaneously occurring inflammation [11,21,26,27,37], and the variation in cytological features in different histological areas of the same lump. These causes lead to mistakes in sample interpretation.

Many tumors fell into a category of cytological grade 1, while in HP they turned out to be grade 2, and hence resulted in the lowest sensitivity of Robinson’s method for cytological grade 2. Like in HBC studies, the mentioned situation and any discordance between cytological and histological gradings may result from assessing different features in both grading systems. For example, the tubule formation and mitotic counts are a fundamental part of the histological grading system, but are not assessed in cytological smears. Many nuclear features are included in the cytological grading, whereas nuclear pleomorphism is the sole criterion in the histological grading [11,15,21,27]. Additionally, the distribution of cases, and heterogeneous morphology are common pitfalls in CMTs, which have an important impact on the final diagnosis.

In accurate diagnosing of benign vs. malignant mammary tumors, cytological samples should be evaluated at both low and high magnification. Our results demonstrated useful and reliable criteria in differentiating benign from malignant mammary tumors, such as cell dissociation, nuclear size, cell uniformity, nucleoli, nuclear margin, chromatin pattern, cellularity, presence of necrotic debris, inflammation, and RBC. This finding was in line with previous published studies in veterinary and human medicine [8,21,22,25]. In contrast, Yildirim and Gurel [25] demonstrated that inflammatory cells, including macrophages and necrotic debris, were not distinctive in making a differentiation between benign and malignant CMTs. Only the evaluation of mucosecretory material and ECM was not sufficient to diagnose the behavior of mammary tumors. The results concerning the presence of ECM corroborate the findings of other authors and support the opinion that the cytological diagnosis of complex/mixed tumors is more challenging and difficult [6,21,37]. However, some studies have reported that the mucosecretory material was much more frequent in the malignant compared to the benign mammary tumors [6,22]

The present study revealed that the cytological features in the modified Robinson’s grading system (cell dissociation, nuclear size, cell uniformity, nucleoli, nuclear margin and chromatin pattern) were positively correlated with histopathological grading of CMTs. Obviously, they were also correlated with the modified Robinson’s grading system for CMTs, which indirectly confirms the high accuracy of this scale. Other parameters, such as the presence of necrotic debris, inflammation, and RBC, were correlated with CP as well as HP grading, whereas the presence of ECM was negatively correlated. Analyses on cellularity and mucosecretory material revealed no significant correlation with the grading. Such results have not been demonstrated previously in CMTs.

Some authors believe that the degree of cell dissociation reflects the tubule formation, which is one of the features considered for HP grading [15]. In our study, cell dissociation corresponded more closely to the grading than to the cellularity of the specimen.

Similarly to our findings, Khan et al. [50] reported a correlation between cellular pleomorphism, nuclear size, nuclear margin, nucleoli, with histological grades in FNAC of HBC. Although in some studies the presence of abnormal mitotic figures was established as an indicator of malignancy in cytological smears of HBC [40,50], in previous reports about CMTs this cytological finding was considered as hindrance due to its infrequency [21,25]. Furthermore, for the same reason Yildirim and Gurel [25] considered the presence of multinucleated giant cells and pseudosyncytial clusters as unimportant criteria in cytological specimens.

Cytomorphometry has been previously used to emphasize the diagnostic importance of CMTs [51,52] as well as HBC [18]. Previous veterinary research demonstrated that NR, MND, and MNA could be used for differentiation of benign from malignant CMTs on cytological smears [6,52]. Although in our study malignant tumors and tumors of grade 2 and 3 based on CP and HP showed higher values of cytomorphometric parameters, these results were not statistically significant. To the best of our knowledge, this study is the first attempt to analyze cytomorphometric parameters in association with the Robinson’s grading of CMTs.

As far as we know, we were the first to evaluate the association between CP grading, cytologic features, metastases, and clinical outcome during the follow-up period. In this study, the high-grade tumors, simple carcinomas diagnosed, and prominent necrotic debris diagnosed in the cytological samples have reflected the metastatic potential of these tumors. The multivariate analysis confirmed that dogs with the tumors of 2 or 3 grade in the cytological examination were at a significantly higher risk of developing metastases compared to the dogs with tumors of grade 1. Regarding the cytological grading, similar results were reported in women [16,44,53].

The presence of tumor necrosis in the histopathological samples has been correlated with malignant behavior, increased histological grade, incidence of lymph node metastases, and decreased survival rate in HBC [54,55] as well in CMTs [56]. Taniguchi et al. [53] proposed considering the presence of necrosis as one of cytological parameters. Consistent with this, a background containing necrotic cellular debris was seen in the majority of malignant mammary tumors [22,53]. Furthermore, some HBC studies have shown the association between increase in cell dissociation and incidence of lymph node metastasis [57]. In contrast, our study has failed to demonstrate cell dissociation in cytologic specimens as a predictive factor of metastases, which was consistent with previously published reports [58]. However, this finding remains an important and appreciated cytological feature included in the Robinson’s grading system.

In general, dogs with a simple type of mammary gland carcinomas are believed to have worse prognosis [11,59], which is consistent with our results regarding CP examination. As we mentioned previously, the agreement between cytological and histopathological types was substantial, and thus may enhance the prognostic value of CP.

Adding cytological grade to cytological diagnosis will provide valuable information about prognosis [15,17,49,53,60]. In the present study, CMTs of grade 2 or 3 in CP were linked with tumor-related cause of death and shorter survival time. Multivariable analysis demonstrated that the cytological grade 2 or 3 and high clinical TNM stage were the only factors that retained statistical significance as independent predictors of shorter survival. Interestingly, cellularity and cytological background features had no prognostic influence on OS. This is a new observation regarding the use of cytological grading of CMTs for prognostic purposes. Further work is required, however, to validate our results. So far only one study [9] has found the association between cytological diagnosis (benign vs. malignant) and postoperative outcome. According to the previous reports on CMTs, we confirmed that in several cases the clinicopathological factors were also significantly associated with metastases and/or survival time after surgery [14,27,56,61].

In summary, our research is the first report evaluating the prognostic value of the modified Robinson’s grading system applied in CMTs. The results indicated the feasibility of using the same cytological criteria established for grading only one type of human breast cancer (i.e. IDC, NOS) for the cytological grading of malignant CMTs regardless their histological type. The data from the current study confirm that various cytological features (included in Robinson’s system and background features) were found to be useful and should be taken into consideration when assessing tumor behavior. The pre-operative availability of cytological examination raises its potential as a diagnostic tool for assessing prognosis. For obvious reasons, histopathology has a strong, indisputable importance for a definitive diagnosis, and therefore due to the limited number of cytopathological studies focusing on prognosis for CMTs further investigations are required on a larger series of cases.

## Supporting information

S1 Table Values of cytomorphometric parameters in benign and malignant tumors according to histopathology. (DOCX)

S2 Table Cytomorphometric parameters in benign tumors/ tumors of grade 1 and tumors of grade 2/ grade 3 according to the Robinson's cytological grading. (DOCX)

S3 Table Comparison of cytomorphometric parameters between benign tumors/ tumors of grade 1 and tumors of grade 2/grade 3 by histopathology as well as between three different grades based on histopathology and cytology, respectively. (DOCX)

S4 Table Evaluation of clinicopathological and histopathological features as risk factors for development of metastasis 2 years after the mastectomy by univariate method. (DOCX)

S5 Table Univariate analysis of clinicopathological and histopathological features affecting the overall survival. (DOCX)
