# Supplementary material for: Diagnostic efficacy of smear cytology and Robinson’s cytological grading of canine mammary tumors with respect to histopathology, cytomorphometry, metastases and overall survival
Source: PLoS One. 2018 Jan 23;13(1):e0191595. doi: 10.1371/journal.pone.0191595 (PMC5779680; doi:10.1371/journal.pone.0191595)
Supplement: S1 Table — (DOCX) [file pone.0191595.s001.docx]

**S1 Table**. **Values of cytomorphometric parameters in benign and malignant tumors according to histopathology.**

| Parameters | Histopathology (Median, IQR, R) | | P-value ^a^ | AUC under ROC curve  (CI 95%) |
| --- | --- | --- | --- | --- |
|  | Benign  n=18 | Malignant  n=55 |  |  |
| MCA (μm^2^) | 133.5,  111.5-159.7, 102.1-851.8 | 151.9,  122.2-168.0,  74.2-360.8 | 0.334 | 57.7%  (41.7%, 73.7%) |
| MCP (μm) | 41.5,  37.3-45.4,  36.0-59.6 | 43.9,  39.3-46.0,  31.5-62.1 | 0.291 | 58.4%  (42.2%, 74.6%) |
| MCD (μm) | 13.1,  11.7-14.2,  11.4-18.7 | 13.8,  12.4-14.3,  9.9-19.5 | 0.353 | 57.4%  (41.2%, 73.5%) |
| MNA (μm^2^) | 63.5,  59.1-75.1,  44.9-106.1 | 74.8,  64.0-82.3,  42.1-206.9 | 0.057 | 65.1%  (50.8%, 79.3%) |
| MNP (μm) | 31.3,  30.2-33.7,  26.2-40.4 | 33.7,  30.9-35.4,  25.7-53.1 | 0.126 | 62.1%  (47.5%, 76.8%) |
| MND (μm) | 8.8,  8.5-9.5,  7.4-11.4 | 9.5,  8.7-10.0,  4.5-15.2 | 0.123 | 62.2%  (48.3%, 76.1%) |
| NR | 1.26,  1.23-1.28,  1.20-1.32 | 1.24,  1.23-1.26,  1.19-1.32 | 0.072 | 64.2%  (49.1%, 79.3%) |
| N/C | 0.55,  0.53-0.60,  0.45-0.63 | 0.59,  0.55-0.65,  0.46-0.93 | 0.087 | 63.9%  (49.5%, 78.3%) |

a – the Mann-Whitney U test, IQR – interquartile range, R – range, MCA – mean cellular area, MCP – mean cellular perimeter, MCD – mean cellular diameter, MNA– mean nuclear area, MNP – mean nuclear perimeter, MND – mean nuclear diameter, NR – nuclear roundness, N/C nuclear to cytoplasmic ratio.
