# Supplementary material for: Diagnostic efficacy of smear cytology and Robinson’s cytological grading of canine mammary tumors with respect to histopathology, cytomorphometry, metastases and overall survival
Source: PLoS One. 2018 Jan 23;13(1):e0191595. doi: 10.1371/journal.pone.0191595 (PMC5779680; doi:10.1371/journal.pone.0191595)
Supplement: S2 Table — (DOCX) [file pone.0191595.s002.docx]

**S2 Table. Cytomorphometric parameters in benign tumors/ tumors of grade 1 and tumors of grade 2/ grade 3 according to Robinson's cytological grading.**

| Parameters | Cytopathology (Median, IQR, R) | | P-value ^a^ | AUC under ROC curve  (CI 95%) |
| --- | --- | --- | --- | --- |
|  | Benign/1 grade  n=41 | 2 and 3 grade  n=32 |  |  |
| MCA (μm^2^) | 135.5,  117.8-162.2,  80.1-851.8 | 151.3,  122.9-166.5, 74.2-321.2 | 0.466 | 55.0%  (41.5%, 68.5%) |
| MCP (μm) | 41.9,  38.8-45.4,  32.1-59.6 | 44.0,  39.7-45.9, 31.5-60.1 | 0.427 | 55.5%  (42.0%, 69.0%) |
| MCD (μm) | 13.2,  12.2-14.3,  10.1-18.7 | 13.8,  12.5-14.3,  9.9-19.5 | 0.460 | 55.1%  (41.6%, 68.6%) |
| MNA (μm^2^) | 68.5,  59.3-80.1,  44.9-194.0 | 75.8,  65.8-82.6, 42.1-206.9 | 0.163 | 59.6%  (46.2%, 73.0%) |
| MNP (μm) | 32.2, 30.3-34.5,  26.2-44.9 | 33.9,  31.2-35.5, 25.7-53.1 | 0.160 | 59.7%  (46.2%, 73.1%) |
| MND (μm) | 9.1,  8.5-9.9,  4.5-12.8 | 9.6,  8.8-10.0,  7.1-15.2 | 0.088 | 61.7%  (48.6%, 74.9%) |
| NR | 1.24,  1.23-1.26,  1.19-1.32 | 1.24,  1.23-1.27,  1.20-1.29 | 0.969 | 49.7%  (36.3%, 63.0%) |
| N/C | 0.58,  0.53-0.62,  0.45-0.75 | 0.59,  0.56-0.65, 0.47-0.93 | 0.120 | 60.8%  (47.6%, 73.9%) |

a – the Mann-Whitney U test, IQR – interquartile range, R – range, MCA – mean cellular area, MCP – mean cellular perimeter, MCD – mean cellular diameter, MNA– mean nuclear area, MNP – mean nuclear perimeter, MND – mean nuclear diameter, NR – nuclear roundness, N/C nuclear to cytoplasmic ratio.
