# Supplementary material for: Diagnostic efficacy of smear cytology and Robinson’s cytological grading of canine mammary tumors with respect to histopathology, cytomorphometry, metastases and overall survival
Source: PLoS One. 2018 Jan 23;13(1):e0191595. doi: 10.1371/journal.pone.0191595 (PMC5779680; doi:10.1371/journal.pone.0191595)
Supplement: S3 Table — (DOCX) [file pone.0191595.s003.docx]

**S3 Table. Comparison of cytomorphometric parameters between benign tumors/ tumors of grade 1 and tumors of grade 2/grade 3 by histopathology as well as between three different grades based on histopathology and cytology, respectively.**

| Parameters | Histopathology | | Cytopathology |
| --- | --- | --- | --- |
|  | Benign/grade 1  vs. 2/3 | Grade 1, 2, 3 | Grade 1, 2, 3 |
|  | P-value^a^ | P-value^b^ | P-value^b^ |
| MCA (μm^2^) | 0.599 | 0.796 | 0.937 |
| MCP (μm) | 0.623 | 0.757 | 0.946 |
| MCD (μm) | 0.678 | 0.811 | 0.949 |
| MNA (μm^2^) | 0.190 | 0.227 | 0.528 |
| MNP (μm) | 0.271 | 0.388 | 0.561 |
| MND (μm) | 0.429 | 0.251 | 0.496 |
| NR | 0.569 | 0.295 | 0.478 |
| N/C | 0.229 | 0.197 | 0.383 |

a – the Mann-Whitney U test, b – the Kruskal-Wallis test, MCA – mean cellular area, MCP – mean cellular perimeter, MCD – mean cellular diameter, MNA– mean nuclear area, MNP – mean nuclear perimeter, MND – mean nuclear diameter, NR – nuclear roundness, N/C nuclear to cytoplasmic ratio.
