# Supplementary material for: Diagnostic efficacy of smear cytology and Robinson’s cytological grading of canine mammary tumors with respect to histopathology, cytomorphometry, metastases and overall survival
Source: PLoS One. 2018 Jan 23;13(1):e0191595. doi: 10.1371/journal.pone.0191595 (PMC5779680; doi:10.1371/journal.pone.0191595)
Supplement: S4 Table — (DOCX) [file pone.0191595.s004.docx]

**S4 Table. Evaluation of clinicopathological and histopathological features as risk factors for development of metastasis 2 years after the mastectomy by univariate method.**

| **Hypothesized**  **risk factor** | **Category** | **Metastases 2 years after the mastectomy / all dogs**  **in the category (%)** | **Crude odds ratio (95% confidence interval)** | **P-value** |
| --- | --- | --- | --- | --- |
| Age | - | - | 1.29 (0.98, 1.69) | 0.064 |
| Purebreed | No  Yes | 3 / 12 (25.0)  12 / 45 (26.7) | 1.09 (0.25, 4.72) | 0.907 |
| Tumor size [cm] | - | - | 1.10 (0.97, 1.24) | 0.138 |
| Tumor size >3cm | No  Yes | 7 / 37 (18.9)  8 / 20 (40.0) | 2.86 (0.85, 9.63) | 0.091 |
| TNM | - | - | - | <0.001^a^* |
| TNM ≥2 | No  Yes | 4 / 34 (11.8)  11 / 23 (47.8) | 6.86 (1.83, 25.89) | 0.004* |
| TNM ≥3 | No  Yes | 6 / 43 (14.0)  9 / 14 (64.3) | 11.1 (2.76, 44.68) | 0.001* |
| TNM = 4 | No  Yes | 8 / 50 (16.0)  7 / 7 (100) | -^b^ | <0.001* |
| Ulceration | No  Yes | 10 / 50 (20.0)  5 / 7 (71.4) | 10.00 (1.69, 59.3) | 0.011* |
| Necrosis | No  Yes | 2 / 28 (7.1)  13 / 29 (44.8) | 10.56 (2.10, 53.04) | 0.004* |
| Invasive growth | No  Yes | 5 / 36 (13.9)  10 / 21 (47.6) | 5.64 (1.58, 20.17) | 0.008* |
| Mitotic counts | - | - | - | 0.010^a^* |
| MC≥1 /HPF | No  Yes | 3 / 36 (8.3)  12 / 21 (57.1) | 14.67 (3.40, 63.42) | <0.001* |
| MC≥ 2 /HPF | No  Yes | 10 / 47 (21.2)  5 / 10 (50.0) | 3.70 (0.89, 15.35) | 0.071 |
| IMC | No  Yes | 11 / 53 (20.8)  4 / 4 (100) | -^b^ | 0.004* |
| HP grade | - | - |  | <0.001^a^* |
| HP grade ≥2 | No  Yes | 1 / 27 (3.7)  14 / 30 (46.7) | 22.75 (2.73, 189.96) | 0.004* |
| HP grade =3 | No  Yes | 4 / 39 (10.3)  11 / 18 (61.1) | 13.75 (3.38, 55.92) | <0.001* |
| Recurrence | No  Yes | 11 / 52 (21.2)  3 / 4 (75) | 11.18 (1.06, 118) | 0.045* |

P-value (*) significant at a significance level (α) of 0.05, a – the Mann-Whitney U test, b – can not be computed when it is 0 or all in one of the category; CMT – canine mammary tumor; TNM – stage 1 (T1N0M0), stage 2 (T2N0M0), stage 3 (T3N0M0), stage 4 (TanyN1M0); MC – mitotic counts, 1. 0-0.9/HPF; 2. 1.0-1.9/HPF; 3. ≥ 2.0 in HPF; HPF – high-power fields (with 40x objective, a 10x eyepiece, FN 22 (field of number of ocular), providing a field area of 0.239mm^2^;

IMC – inflammatory mammary carcinoma.
