# Supplementary material for: Diagnostic efficacy of smear cytology and Robinson’s cytological grading of canine mammary tumors with respect to histopathology, cytomorphometry, metastases and overall survival
Source: PLoS One. 2018 Jan 23;13(1):e0191595. doi: 10.1371/journal.pone.0191595 (PMC5779680; doi:10.1371/journal.pone.0191595)
Supplement: S5 Table — (DOCX) [file pone.0191595.s005.docx]

**S5 Table. Univariate analysis of clinicopathological and histopathological features affecting the overall survival.**

| **Hypothesized prognostic factor** | **Category** | **CMT-related deaths / all dogs in the category (%)** | **Hazard ratio (95% confidence interval)** | **P-value** |
| --- | --- | --- | --- | --- |
| Age | - | - | 1.46 (1.13, 1.88) | 0.004* |
| Tumor size [cm] | - | - | 1.08 (0.99, 1.18) | 0.068 |
| TNM | - | - | - | <0.001^a^* |
| TNM ≥2 | No  Yes | 3 / 33 (9.1)  11 / 26 (42.3) | 5.33 (1.48, 19.16) | <0.010* |
| TNM ≥3 | No  Yes | 5 / 45 (11.1)  9 / 14 (64.3) | 10.25 (3.13, 33.55) | <0.001* |
| TNM = 4 | No  Yes | 7 / 52 (13.5)  7 / 1 (100) | 34.17 (9.91, 117) | <0.001* |
| Ulceration | No  Yes | 9 / 52 (17.3)  5 / 7 (71.4) | 5.60 (1.80, 17.45) | 0.003* |
| Necrosis | No  Yes | 2 / 28 (7.1)  12 / 31 (38.7) | 13.83 (1.79, 107) | 0.012* |
| Invasive growth | No  Yes | 4 / 36 (11.1)  10 / 23 (43.5) | 6.35 (1.74, 23.11) | 0.005* |
| Mitotic counts | - |  | - | 0.002^a^* |
| MC ≥1 /HPF | No  Yes | 4 / 38 (10.5)  10 / 21 (47.6) | 7.60 (2.09, 27.73) | 0.002* |
| MC ≥2 /HPF | No  Yes | 9 / 48 (18.8)  5 / 11 (45.5) | 2.97 (0.96, 9.19) | 0.059 |
| IMC | No  Yes | 10 / 55 (18.2)  4 / 4 (100) | 39.73 (6.88, 230) | <0.001* |
| HP grade | - |  |  | 0.002^a^* |
| HP grade ≥2 | No  Yes | 1 / 29 (3.5)  13 / 30 (43.3) | 15.16 (1.98, 116) | 0.009* |
| HP grade =3 | No  Yes | 3 / 37 (8.1)  11 / 19 (57.9) | 15.54 (3.43, 70.55) | <0.001* |
| Recurrence | No  Yes | 10 / 55 (18.2)  4 / 4 (100) | 4.10 (1.18, 14.29) | 0.027* |

P-value (*) significant at a significance level (α) of 0.05, a – generalized Mantel-Cox log rank test; TNM – stage 1 (T1N0M0), stage 2 (T2N0M0), stage 3 (T3N0M0), stage 4 (TanyN1M0); MC – mitotic counts, 1. 0-0.9/HPF; 2. 1.0-1.9/HPF; 3. ≥ 2.0 in HPF; HPF – high-power fields (with 40x objective, a 10x eyepiece, FN 22 (field of number of ocular), providing a field area of 0.239mm^2^, IMC – inflammatory mammary carcinoma.
